# Healthcare Utilisation in Ageing Adults With Intellectual Disability: Longitudinal Evidence From Five Waves of IDS‐TILDA

**DOI:** 10.1111/jar.70236

**Published:** 2026-04-28

**Authors:** Martin McMahon, Aviejay Paul, Catriona Ryan, Louise Lynch, Philip McCallion, Mary McCarron, Eilish Burke

**Affiliations:** ^1^ School of Nursing and Midwifery, Trinity Centre for Ageing and the Life Course in Intellectual Disability Trinity College Dublin Ireland; ^2^ Biostatistics Unit, Discipline of Public Health and Primary Care, School of Medicine Trinity College Dublin Dublin Ireland; ^3^ Wellcome‐HRB Clinical Research Facility St James's Hospital Dublin Ireland; ^4^ School of Social Work, Temple University Barnett College of Public Health Philadelphia Pennsylvania USA

**Keywords:** healthcare utilisation, intellectual disability, multimorbidity, residence

## Abstract

**Background:**

Adults with intellectual disability are living longer and ageing with more illness. This impacts healthcare utilisation. Yet little is known about how healthcare utilisation changes over time as this population ages.

**Methods:**

Five waves of longitudinal data of people ≥ 40 years with intellectual disability from 2009 to 2023 were analysed. Generalised linear multilevel models examined associations between sociodemographic characteristics and multimorbidity with patterns of General Practitioner (GP), outpatient and emergency department use.

**Results:**

GP use was consistently high across all waves. Outpatient use increased as people aged, while emergency department use was associated with age greater than 65, multimorbidity and conditions such as neurological, joint, gastrointestinal, endocrine and heart disease. Residence was a significant factor influencing healthcare utilisation.

**Conclusion:**

Our findings demonstrate that multimorbidity, residence and age were associated with healthcare utilisation. These are important insights to help support the planning of accessible and preventative health services for this population.

## Summary

- GP use was consistently high, with outpatient use increasing as people aged. Emergency department use was associated with multimorbidity and older age.
- Where people live had an impact on how much healthcare they used.
- These findings point to gaps in access that need attention as people get older.
- This study provides evidence that can help improve how services are planned and delivered for ageing adults with intellectual disability.

## 1 Background

Adults with intellectual disability are living longer, reflecting significant advances across healthcare. Yet people with intellectual disability still die at younger ages (Dolan et al. 2021), often from preventable causes (Heslop et al. 2013; Lauer and McCallion 2015; Thygesen et al. 2024) and on average around 20 years earlier than the general population (McCarron et al. 2015; O'Leary et al. 2018). While causes of death are similar to the general population, the hierarchy of primary causes differs. Respiratory disease is the leading cause of death in the intellectual disability population, whereas cardiovascular disease and cancer are main causes of death in the general population in high‐income countries (Heslop et al. 2013; McMahon et al. 2023; World Health Organisation 2024).

Life expectancy has increased but with little compression of morbidity with the age of onset of most health problems not increasing in parallel with life expectancy (Crimmins 2015; Permanyer et al. 2023). This is even more pronounced for people with intellectual disability, with complex health profiles (Fitzpatrick et al. 2025) developing at younger ages (McMahon and Hatton 2021). This reflects greater exposure to social determinants of health (Chapman et al. 2024) and associated underlying conditions (Lewis and Bench 2025), with examples including neuropathological changes and Alzheimer's‐type dementia beginning much earlier in adults with Down syndrome (McCarron et al. 2014), as well as epilepsy (Snoeijen‐Schouwenaars et al. 2021), thyroid disorders (Liao et al. 2021), diabetes (Baksh et al. 2025) and musculoskeletal disorders (de Leeuw et al. 2025; Liao et al. 2021; McMahon and Hatton 2021).

Multimorbidity (≥ 2 co‐existing conditions) is a growing global challenge for ageing populations (Skou et al. 2022). It is associated with increased healthcare utilisation in the general population (Palladino et al. 2016; Soley‐Bori et al. 2020) and among people with intellectual disability (Ryan et al. 2025). Nonetheless, healthcare utilisation for people with intellectual disability is often described as fragmented, exclusionary, reactive, poorly coordinated and not meeting this population's needs (Ali et al. 2013; Cooper et al. 2018; Doherty et al. 2020; Shady et al. 2022; Tuffrey‐Wijne et al. 2014). Additionally, those with intellectual disability who are not known to services are more likely to encounter greater challenges in healthcare utilisation (Emerson and Hatton 2014). Consequently, many people with intellectual disability do not receive equitable healthcare across the continuum of diagnosis, management and ongoing treatment (Chapman et al. 2024). Evidence frequently highlights that people with intellectual disability also often receive poorer quality of care (compared to the general population) across primary, secondary and tertiary services (Baksh et al. 2021; Hanlon et al. 2018; Hansford et al. 2025; van den Bemd et al. 2024). This is unjust, unfair, wholly avoidable and reflective of systemic and practice inequities (Emerson and Hatton 2014; McMahon et al. 2024).

How healthcare utilisation and healthcare needs for people with intellectual disability intersect, and change over time, is not well researched. Nor is how healthcare utilisation is influenced by socio‐economic circumstances, educational attainment and health literacy, as well as the accessibility and availability of services (Kreps et al. 2025; Ng et al. 2024). Instead, healthcare utilisation research has primarily been cross‐sectional (Maltais et al. 2020; McCarron et al. 2017), lacking clinical data (Reppermund et al. 2019) and focused on single attendance (Carey et al. 2016) or specific conditions (Liao et al. 2022; Lu et al. 2020). Longitudinal studies which track individuals with intellectual disability offer a unique opportunity to explore the trajectories of changing sociodemographic and healthcare needs and how they influence patterns of healthcare utilisation.

In Ireland, healthcare services for people with intellectual disabilities are provided through a mix of public and voluntary, not‐for‐profit organisations. Access is typically supported to GPs and specialist dental, optical, and aural services by public coverage schemes (e.g., medical cards) meaning there is no cost to eligible participants. Secondary services are generally provided through mainstream hospitals and costs are often exempt for medical card holders. From this perspective, our study uses longitudinal data to examine trends in healthcare utilisation and health conditions among older Irish adults with intellectual disability with a particular focus on how sociodemographic and clinical characteristics are associated with healthcare utilisation over time.

## 2 Methods

### 2.1 Study Design and Setting

This is a secondary analysis of longitudinal data from the Intellectual Disability Supplement to The Irish Longitudinal Study on Ageing (IDS‐TILDA), a nationally representative longitudinal cohort study of adults with intellectual disability aged ≥ 40 years living in Ireland. Further methodological details are available elsewhere (McCarron et al. 2023).

### 2.2 Participants

IDS‐TILDA consists of five interview waves: Wave 1 (2009–2010), Wave 2 (2013–2014), Wave 3 (2016–2017), Wave 4 (2019–2020) and Wave 5 (2022–2023). Of a nationally representative cohort of 753 adults aged ≥ 40 with intellectual disability who participated at Wave 1, 708 participants remained in Wave 2, 609 in Wave 3, and 506 in Wave 4. Losses due to death, loss to follow‐up, and a small number of withdrawals led to recruiting 233 new participants at Wave 4 (739 total participants), and further losses by Wave 5 led to the addition of 141 new participants (762 total participants).

### 2.3 Ethics Approval

IDS‐TILDA has been granted full ethical approval for all waves, and a consent declaration was granted by the Health Research Consent Declaration Committee since its inception in 2019.

### 2.4 Variables

#### 2.4.1 Outcome Variables

This study explored the effect of chronic conditions and multimorbidity on longitudinal trends in the utilisation of GP, outpatient and emergency department services, controlling for demographic predictors. Utilisation was treated as a binary ‘yes/no’ variable. The combinations of diseases that make up each chronic health condition are outlined in Table 1. The presence of two or more overall chronic health conditions was classified as multimorbidity. All outcomes variables were self‐or proxy‐reported without clinical verification and represent the condition at that wave.

**TABLE 1:** Disease combination for each condition.

Condition	Diseases combined in all waves
Hypertension	Hypertension
Eye disease	Age‐related macular degeneration, glaucoma, cataract, keratoconus, other eye disease, cataract surgery
Heart disease	Heart murmur, abnormal heart rhythm, angina, heart attack, angioplasty or stent, congestive heart failure, open heart surgery, other heart disease
Endocrine disease	Diabetes, thyroid
Joint disease	Arthritis, osteoporosis, scoliosis, rheumatoid arthritis, osteoarthritis, other kind of arthritis.
Lung disease	Asthma, other lung disease
Gastrointestinal disease	Constipation, coeliac disease, gastroesophageal disease, phenylketonuria, stomach ulcer
Mental health condition	Depression, schizophrenia, psychiatric condition, hallucinations, anxiety, emotional problems, mood swings, manic depression, psychosis, others.
Stroke	Stroke and TIA
Cancer	Cancer
Neurological disease	Cerebral palsy, epilepsy, multiple sclerosis, Parkinson's disease, spina bifida, muscular dystrophy, Alzheimer's, dementia (combined with Alzheimer's in W4 CAPI), memory impairment
Liver disease	Serious liver damage (cirrhosis)

#### 2.4.2 Predictor Variables

Five predictor variables were used:

1. Sex: female or male; reference: male
2. Age: less than 50, 50–64, 65+; reference: less than 50
3. Level of intellectual disability: mild, moderate, severe/profound; reference: mild
4. Down syndrome: did not report Down syndrome, reported Down syndrome; reference: did not report Down syndrome
5. Residential setting: (fixed) living independently/with family, community group home (CGH), residential setting; reference: living independently/with family

### 2.5 Statistical Analysis

Descriptive statistics of the demographic predictors were generated and longitudinal trends in the healthcare utilisation of different medical services across the five waves were visualised using line graphs. As this was a secondary analysis of longitudinal data with fixed sample sizes, a priori power calculation was not undertaken. Intraclass correlation coefficients (ICCs) were calculated from unconditional mean models to determine whether there was enough variation between the clusters to use multilevel modelling. The unconditional mean models were also used to estimate the mean odds of utilising healthcare services across the waves (Sommet and Morselli 2017). Generalised linear mixed models (GLMMs) were fitted to analyse the longitudinal relationships between predictors and each healthcare utilisation outcome. Repeated observations were clustered within participants across waves to account for the non‐independence of repeated measurements from the same individual. A *p* value of 0.05 was used to identify statistically significant associations. GLMM models identified whether different chronic conditions affected trends in utilisation of services across five waves, controlling for participant demographic characteristics. Non‐linear effects of time were modelled using second and third‐order polynomial terms of the wave variable. Effects were compared with a linear specification of time using the Akaike information criterion (AIC) and Bayesian information criterion (BIC). Additionally, time was examined as a categorical variable. The temporal functional form that minimised the AIC and BIC values in the unadjusted models was retained for the adjusted analyses. The number of adaptive Gauss–Hermite quadrature points (nAGQ) was further optimised to ensure the numerical stability and accuracy of parameter estimation. GLMM results are presented as adjusted odds ratios (ORs) with 95% confidence intervals. All statistical analyses were conducted using R.

## 3 Results

**FIGURE 1:**
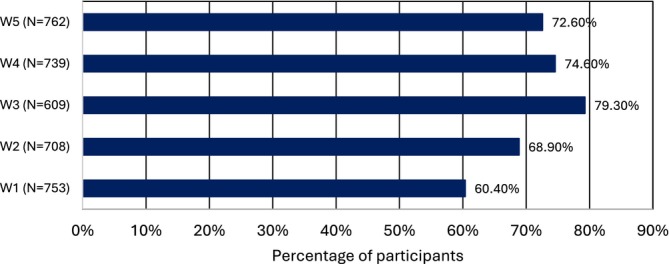
Prevalence of multimorbidity across IDS‐TILDA waves 1–5.

**FIGURE 2:**
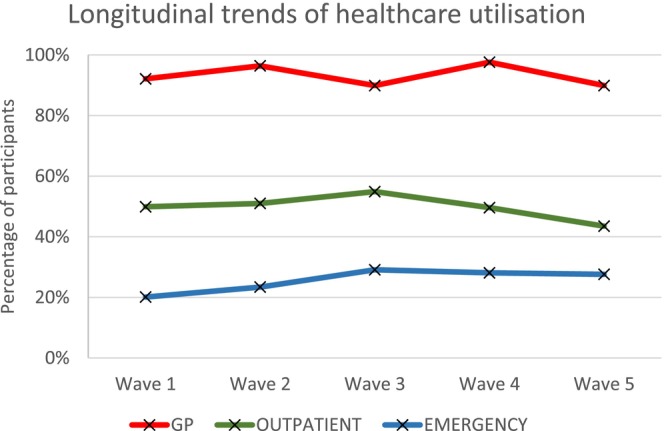
Longitudinal trends in healthcare utilisation.

**FIGURE 3:**
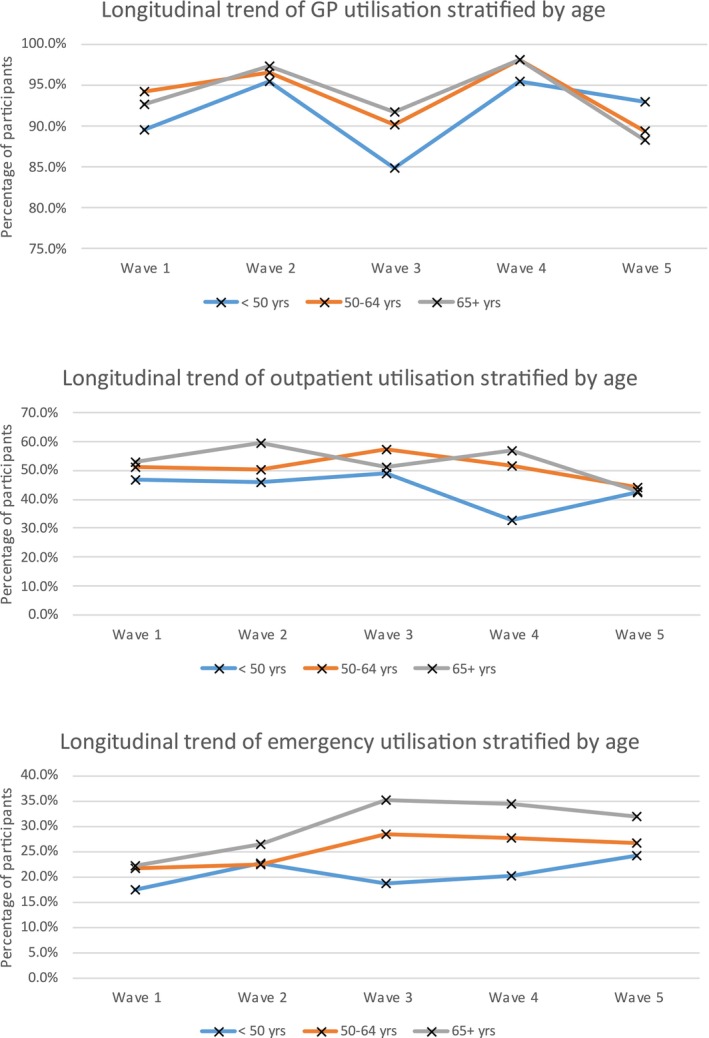
Longitudinal trends in healthcare utilisation stratified by age.

Descriptive statistics are outlined in Table 2. Figure 1 shows the prevalence of multimorbidity over the five waves. Figure 2 shows GP utilisation over the five waves was the highest, followed by outpatient services and emergency services, respectively. Figure 3 shows a similar pattern stratified by age. GP use remained high across all groups and age‐related increases were visible for emergency services.

**TABLE 2:** Descriptive statistics of demographic predictors across 5 waves.

	W1 (*N* = 753)	W2 (*N* = 708)	W3 (*N* = 609)	W4 (*N* = 739)	W5 (*N* = 762)
	*n*, %	*n*, %	*n*, %	*n*, %	*n*, %
Gender					
Male	335, 44.5	312, 44.1	269, 44.2	344, 46.5	355, 46.6
Female	418, 55.5	396, 55.9	340, 55.8	395, 53.5	407, 53.4
Age					
< 50	288, 38.2	197, 28.1	78, 11.8	137, 18.5	183, 24.0
50–64	343, 45.6	356, 50.8	381, 62.6	408, 55.2	363, 47.6
65+	122, 16.2	148, 21.1	156, 25.6	194, 26.3	216, 28.3
Level of intellectual disability					
Mild	168, 24.0	158, 24.2	139, 24.8	179, 27.0	205, 29.8
Moderate	323, 46.5	304, 46.5	259, 46.2	298, 45.0	312, 45.3
Severe/profound	205, 29.5	192, 29.4	163, 29.1	185, 27.9	172, 25.0
Down syndrome					
Did not report Down syndrome	603, 80.1	567, 80.1	497, 81.6	593, 80.2	621, 81.5
Reported Down syndrome	150, 19.9	141, 19.9	112, 18.4	146, 19.8	141, 18.5
Residence					
Independent/family	129, 17.1	113, 16.2	95, 15.6	126, 17.3	172, 22.6
CGH	268, 35.6	303, 43.3	246, 40.4	358, 49.0	373, 49.0
Residential setting	356, 47.3	283, 40.5	268, 44.0	246, 33.7	217, 28.5

Mean odds describe the average likelihood of reporting any service use (yes/no) across all repeated observations. The odds of GP utilisation were 15.4 (95% CI: 12.1–19.6), indicating utilisation was far more common than non‐utilisation. Emergency department utilisation odds were 0.295 (95% CI: 0.264–0.330), meaning non‐utilisation was more common than utilisation (Table 3). Participants were equally likely to use outpatient services as not, with odds close to 1 (0.959).

**TABLE 3:** Mean odds of healthcare utilisation based on repeated measures across all five IDS‐TILDA waves.

Healthcare utilisation	Mean odds of healthcare utilisation across 5 waves (95% CI) $\;\frac{P\left(X=1\right)}{P\left(X=0\right)}$
GP	15.4 (12.1–19.6)
Outpatient	0.959 (0.868–1.06)
ED	0.295 (0.264–0.33)

The intraclass correlation coefficient (ICC) calculated from the unconditional mean model for GP was 0.085, while the ICC for the outpatient model was 0.225 and that for emergency department services was 0.169, meaning there was enough data clustering to warrant using GLMMs. When the GLMM models were adjusted for the demographic predictors, results showed that participants living in community group homes were almost twice as likely to use GP and outpatient services as those who lived independently or with family, while such participants were 1.47 (1.07–2.02) times more likely to avail of emergency services (see Table 4). Overall, participants living in residential settings were even more likely to avail of these healthcare services. Participants in the age category 50–64 years were 1.26 (1.01–1.58) times more likely to use outpatient services compared to participants whose age was less than 50 years, while participants who were 65+ years of age were 1.5 (1.10–2.03) times more likely to use emergency services than those less than 50 years of age. Neither level of intellectual disability nor Down syndrome status significantly influenced healthcare utilisation.

**TABLE 4:** Adjusted odds ratios (ORs) and 95% confidence intervals from generalised linear mixed models for GP, outpatient, and emergency department utilisation across five IDS‐TILDA waves.

Variables	GP services	Outpatient services	Emergency department services
GENDER = female	1.145 (0.846–1.550); 0.381	1.105 (0.899–1.358); 0.343	1.062 (0.8586–1.313); 0.581
GENDER = male	Reference category		
AGE_CAT = 65+	1.054 (0.661–1.682); 0.824	1.322 (0.984–1.776); 0.064	1.499 (1.1020–2.038); 0.010
AGE_CAT = 50–64	1.108 (0.766–1.603); 0.586	1.262 (1.010–1.577); 0.041	1.196 (0.9377–1.526); 0.149
AGE_CAT < 50	Reference category		
Level of intellectual disability = severe/profound	1.009 (0.635–1.603); 0.971	0.861 (0.638–1.161); 0.326	0.846 (0.6219–1.151); 0.287
Level of intellectual disability = moderate	0.870 (0.595–1.271); 0.472	0.861 (0.665–1.114); 0.255	0.845 (0.6475–1.103); 0.215
Level of intellectual disability = mild	Reference category		
Down syndrome = yes	0.872 (0.596–1.275); 0.480	1.185 (0.911–1.541); 0.207	1.126 (0.8601–1.475); 0.387
Down syndrome = no	Reference category		
RESIDENCE = residential setting	1.596 (1.020–2.498); 0.041	2.176 (1.586–2.985); < 0.001	1.928 (1.3721–2.709); < 0.001
RESIDENCE = CGH	1.920 (1.272–2.898); 0.002	1.926 (1.441–2.574); < 0.001	1.474 (1.0741–2.022); 0.016
RESIDENCE = independent/family	Reference category		
Wave 5	0.773 (0.512–1.166); 0.219	Wave used as continuous variable	Wave used as continuous variable
Wave 4	3.222 (1.681–6.178); < 0.001	Wave used as continuous variable	Wave used as continuous variable
Wave 3	0.628 (0.412–0.957); 0.030	Wave used as continuous variable	Wave used as continuous variable
Wave 2	2.144 (1.276–3.602); 0.004	Wave used as continuous variable	Wave used as continuous variable
Wave 1	Reference category	Wave used as continuous variable	Wave used as continuous variable
Wave	Wave used as categorical variable	1.107 (0.962–1.273); 0.158	1.286 (1.104–1.499); 0.001
$\mathrm{Wav}e^{3}$	Wave used as categorical variable	0.990 (0.982–0.998); 0.020	0.993 (0.984–1.001); 0.096

When the GLMM models controlled for the chronic conditions as shown in Tables S1–S3, it was found that none of the conditions were significantly associated with utilisation of GP services across any of the five waves. Participants who had neurological disease, joint disease, gastrointestinal disease and stroke were more likely to use emergency services. Participants with multimorbidity were 1.64 (1.29–2.08) times more likely to use emergency services than those who were not multimorbid. Participants with neurological disease, heart disease, endocrine disease, joint disease, gastrointestinal disease, cancer and multimorbidity (Figure 1) were more likely to use outpatient services compared to those without these conditions.

## 4 Discussion

Collectively, our brief report findings indicate that GP use is high and stable across all waves with outpatient use associated with chronic disease burden such as multimorbidity and neurological, cardiac, endocrine, joint, gastrointestinal, and cancer‐related conditions, even when the impact of the COVID‐19 pandemic is considered. Minor changes in utilisation levels during the pandemic (Wave 4) may indicate a prioritisation and/or reorganisation of care for this group at this timepoint for this population and this is potentially reflected in the low levels of mortality from COVID‐19 observed in this population in Ireland at this wave (McMahon et al. 2023). Emergency department use is associated with neurological disease, joint disease, gastrointestinal disease, and stroke, and among those with multimorbidity, highlighting the responsive and unplanned nature of emergency care and potentially the higher levels of unmet healthcare needs in adults with intellectual disability (Axmon et al. 2025; Liao et al. 2022). As GP use was not associated with disease burden whereas emergency department use was, our findings signal that emergency department use is potentially sensitive to acute complications, or crises related to neurological, joint and gastrointestinal conditions (e.g., seizures, reduced mobility, constipation), which can trigger unplanned care even with high levels of GP use. Increasing use of outpatient services was found in those aged 50–64 and of emergency department attendance in those aged 65 and older further highlighting the relationship of ageing with the rising burden of multimorbidity and functional decline (McCarron et al. 2017, 2023). The higher levels of emergency department and outpatient use observed among participants with multimorbidity highlight the greater service needs associated with complex long‐term conditions. While we cannot determine from this study whether preventative care was provided, the findings underline the importance of strengthening coordinated and accessible models of care to help reduce avoidable escalation to unplanned services.

Results indicate sociodemographic factors influence healthcare utilisation. While results show that living arrangement is one of the strongest predictors of GP use, not disease burden or level of intellectual disability, this may reflect ceiling effects. Importantly, it needs to be considered that high levels of utilisation do not necessarily equate to high quality healthcare provision; rather, it may imply a greater reliance on services or care fragmentation (Kelleher et al. 2025). This is potentially very concerning. For example, there is consistent evidence that those who are unknown to services have similar or worse health (Emerson and Hatton 2014; Emerson et al. 2016) and are exposed to the social determinants that increase poorer health (Marmot 2005). Moreover, a recent review reported formal supports were associated with a greater likelihood of seeing a healthcare provider (Barrington et al. 2025). Consequently, those unknown populations are more likely to be further overlooked in health promotion initiatives, exacerbating exclusion from mainstream services (Barrington et al. 2025) and widening inequities in access (Chapman et al. 2024).

Our study is not without limitations, and it is important to note that chronic conditions relied on self‐ or proxy‐reported data without clinical verification which may introduce bias. Other important aspects of utilisation such as planned or unplanned care were not recorded, which would offer additional insights. A binary (yes/no) utilisation measure limits the capacity to distinguish between one and multiple visits while living arrangements were treated as fixed in the analysis, and these may change over time. Sample refreshes may have introduced some differences not accounted for, but our maintaining population representativeness may have reduced this risk. Finally, Wave 4 coincided with the COVID‐19 pandemic which impacted the delivery and organisation of healthcare services worldwide. This timepoint may represent a period effect and should be considered from this viewpoint. Equally, as the analysis pooled estimates across all waves, this should be interpreted cautiously as averages, rather than assuming stable service availability throughout all waves.

Notwithstanding this, the trajectory of healthcare utilisation will continue to be shaped by increases in longevity for this population. This demands meaningful attention in Ireland and internationally. Existing evidence in this sphere (Axmon et al. 2025; Carey et al. 2016; McCarron et al. 2017) is limited by cross‐sectional analysis and, in some cases, limited to COVID‐19 pandemic specific scope. This study draws on a large, nationally representative longitudinal dataset spanning five waves over 14 years. It goes beyond describing overall utilisation by identifying factors associated with service use, offering deeper insight into patterns of healthcare utilisation in ageing adults with intellectual disability. Key strengths include the large nationally representative sample, the true longitudinal design with extended follow‐up, rich clinical and demographic data, and examination of multiple healthcare settings. As a final caution, it should be accepted that our findings relate to people known to health and social care services and there remains a large cohort of people not known who could be further marginalised and excluded from proactive, preventative healthcare. From an equity perspective, this group must be prioritised.

## Author Contributions

**Martin McMahon:** conceptualisation (lead), methodology (lead), writing – original draft (lead), writing – review and editing (equal). **Aviejay Paul:** formal analysis (lead), review and editing (equal). **Catriona Ryan:** formal analysis (equal), review and editing (equal). **Louise Lynch**, **Philip McCallion** and **Eilish Burke:** conceptualisation (supporting), writing – original draft (supporting), writing – review and editing (equal). **Mary McCarron:** funding acquisition (lead), conceptualisation (supporting), writing – original draft (supporting), writing – review and editing (equal).

## Funding

This research was funded by the Health Research Board Ireland IDS‐TILDA‐2021‐001 and the Department of Children, Disability and Equality (DCDE).

## Conflicts of Interest

The authors declare no conflicts of interest.

## Supporting information


**Table S1:** Odds ratios (OR) and 95% confidence intervals (CI) from GLMM results for utilisation of GP services across 5 waves controlled for the demographic predictors and chronic conditions.
**Table S2:** GLMM results for utilisation of emergency services across 5 waves controlled for the demographic predictors and chronic conditions.
**Table S3:** Odds ratios (OR) and 95% confidence intervals (CI) from GLMM results for utilisation of outpatient services across 5 waves controlled for the demographic predictors and chronic conditions.

## Data Availability

The data that support the findings of this study are available from the corresponding author upon reasonable request.
