# Supplementary material for: Healthcare Utilisation in Ageing Adults With Intellectual Disability: Longitudinal Evidence From Five Waves of IDS‐TILDA
Source: J Appl Res Intellect Disabil. 2026 Apr 28;39:e70236. doi: 10.1111/jar.70236 (PMC13125395; doi:10.1111/jar.70236)
Supplement: Supplementary file 1 — Table S1: Odds ratios (OR) and 95% confidence intervals (CI) from GLMM results for utilisation of GP services across 5 waves controlled for the demographic predictors and chronic conditions. Table S2: GLMM results for utilisation of emergency services across 5 waves controlled for the demographic predictors and chronic conditions. Table S3: Odds ratios (OR) and 95% confidence intervals (CI) from GLMM results for utilisation of outpatient services across 5 waves controlled for the demographic predictors and chronic conditions. [file JAR-39-e70236-s001.docx]

Supplementary Table 1: *Odds ratios (OR) and 95% confidence intervals (CI) from* GLMM results for utilisation of GP services across 5 waves controlled for the demographic predictors and chronic conditions

| **Variable** | **Hypertension** | **Lung disease** | **Mental health** | **Stroke** | **Neurological disease** | **Eye disease** |
| --- | --- | --- | --- | --- | --- | --- |
| GENDER=female | 1.141 (0.844–1.544); 0.391 | 1.142 (0.843–1.546); 0.392 | 1.144 (0.845–1.548); 0.385 | 1.145 (0.846–1.550); 0.381 | 1.145 (0.846–1.550); 0.381 | 1.164 (0.860–1.580); 0.325 |
| GENDER=male | Reference Category | | | | | |
| AGE=65+ | 1.002 (0.624–1.609); 0.993 | 1.036 (0.649–1.653); 0.883 | 1.053 (0.660–1.679); 0.828 | 1.011 (0.633–1.620); 0.962 | 1.054 (0.661–1.682); 0.824 | 1.107 (0.691–1.770); 0.674 |
| AGE=50–64 | 1.084 (0.749–1.570); 0.669 | 1.102 (0.762–1.595); 0.606 | 1.107 (0.765–1.602); 0.588 | 1.101 (0.761–1.590); 0.610 | 1.108 (0.766–1.604); 0.585 | 1.127 (0.778–1.630); 0.527 |
| AGE_CAT < 50 | Reference Category | | | | | |
| Level of intellectual disability =severe/profound | 1.033 (0.650–1.641); 0.892 | 1.023 (0.643–1.627); 0.924 | 1.003 (0.631–1.594); 0.991 | 1.025 (0.644–1.630); 0.918 | 1.009 (0.633–1.610); 0.969 | 1.015 (0.639–1.610); 0.950 |
| Level of intellectual disability =moderate | 0.872 (0.597–1.273); 0.478 | 0.877 (0.600–1.283); 0.500 | 0.867 (0.594–1.268); 0.463 | 0.879 (0.601–1.280); 0.505 | 0.870 (0.595–1.273); 0.474 | 0.876 (0.600–1.280); 0.492 |
| Level of intellectual disability =mild | Reference Category | | | | | |
| Down Syndrome = yes | 0.895 (0.611–1.310); 0.567 | 0.866 (0.592–1.266); 0.457 | 0.887 (0.603–1.304); 0.542 | 0.869 (0.594–1.270); 0.468 | 0.872 (0.596–1.275); 0.480 | 0.925 (0.627–1.360); 0.693 |
| Down Syndrome = no | Reference Category | | | | | |
| RESIDENCE=residential setting | 1.604 (1.026–2.507); 0.038 | 1.584 (1.012–2.479); 0.044 | 1.559 (0.986–2.464); 0.057 | 1.563 (0.998–2.450); 0.051 | 1.597 (1.016–2.510); 0.042 | 1.632 (1.043–2.560); 0.032 |
| RESIDENCE=Community group home | 1.926 (1.278–2.904); 0.002 | 1.915 (1.269–2.889); 0.002 | 1.887 (1.242–2.865); 0.003 | 1.911 (1.267–2.880); 0.002 | 1.920 (1.272–2.900); 0.002 | 1.953 (1.294–2.950); 0.001 |
| RESIDENCE=Independent/family | Reference Category | | | | | |
| Disease = yes | 1.267 (0.813–1.976); 0.296 | 1.454 (0.767–2.754); 0.251 | 1.080 (0.784–1.487); 0.638 | 1.946 (0.691–5.480); 0.208 | 0.997 (0.725–1.371); 0.985 | 0.776 (0.538–1.120); 0.176 |
| Disease = no | Reference Category | | | | | |
| Wave=5 | 0.774 (0.513–1.167); 0.222 | 0.771 (0.511–1.164); 0.216 | 0.772 (0.512–1.166); 0.219 | 0.771 (0.511–1.160); 0.214 | 0.773 (0.512–1.167); 0.220 | 0.789 (0.522–1.190); 0.260 |
| Wave=4 | 3.225 (1.683–6.180); 0.000 | 3.225 (1.682–6.183); 0.000 | 3.216 (1.678–6.166); 0.000 | 3.194 (1.666–6.120); 0.000 | 3.222 (1.681–6.178); 0.000 | 3.269 (1.704–6.270); 0.000 |
| Wave=3 | 0.628 (0.412–0.957); 0.030 | 0.626 (0.411–0.954); 0.030 | 0.627 (0.411–0.955); 0.030 | 0.623 (0.409–0.950); 0.028 | 0.628 (0.412–0.958); 0.031 | 0.636 (0.417–0.970); 0.035 |
| Wave=2 | 2.145 (1.277–3.603); 0.004 | 2.144 (1.276–3.602); 0.004 | 2.141 (1.274–3.597); 0.004 | 2.130 (1.268–3.580); 0.004 | 2.144 (1.276–3.602); 0.004 | 2.143 (1.276–3.600); 0.004 |
| Wave=1 | Reference Category | | | | | |

| **Variable** | **Heart disease** | **Endocrine disease** | **Joint disease** | **Gastro-intestinal disease** | **Cancer** | **Multimorbidity** |
| --- | --- | --- | --- | --- | --- | --- |
| GENDER=female | 1.140 (0.842–1.544); 0.397 | 1.173 (0.864–1.593); 0.307 | 1.162 (0.857–1.577); 0.334 | 1.137 (0.840–1.540); 0.406 | 1.147 (0.847–1.553); 0.375 | 1.123 (0.829–1.521); 0.455 |
| GENDER=male | Reference Category | | | | | |
| AGE=65+ | 1.074 (0.673–1.715); 0.765 | 1.081 (0.676–1.727); 0.745 | 1.082 (0.675–1.733); 0.744 | 1.035 (0.648–1.652); 0.885 | 1.047 (0.656–1.671); 0.847 | 1.010 (0.631–1.618); 0.965 |
| AGE=50–64 | 1.111 (0.767–1.608); 0.578 | 1.129 (0.779–1.636); 0.521 | 1.118 (0.772–1.618); 0.555 | 1.106 (0.764–1.600); 0.593 | 1.107 (0.766–1.602); 0.588 | 1.088 (0.751–1.574); 0.656 |
| AGE_CAT < 50 | Reference Category | | | | | |
| Level of intellectual disability =severe/profound | 1.000 (0.629–1.591); 0.999 | 1.003 (0.631–1.595); 0.989 | 1.014 (0.638–1.611); 0.952 | 0.941 (0.586–1.511); 0.803 | 1.015 (0.638–1.613); 0.951 | 0.974 (0.611–1.553); 0.913 |
| Level of intellectual disability =moderate | 0.876 (0.599–1.282); 0.496 | 0.869 (0.595–1.271); 0.469 | 0.875 (0.599–1.278); 0.489 | 0.848 (0.580–1.241); 0.397 | 0.872 (0.597–1.274); 0.478 | 0.854 (0.584–1.248); 0.414 |
| Level of intellectual disability =mild | Reference Category | | | | | |
| Down Syndrome = yes | 0.902 (0.614–1.323); 0.597 | 0.932 (0.628–1.382); 0.725 | 0.870 (0.595–1.271); 0.472 | 0.880 (0.602–1.286); 0.508 | 0.874 (0.598–1.278); 0.487 | 0.874 (0.598–1.277); 0.487 |
| Down Syndrome = no | Reference Category | | | | | |
| RESIDENCE=residential setting | 1.612 (1.029–2.525); 0.037 | 1.619 (1.033–2.536); 0.035 | 1.616 (1.032–2.530); 0.036 | 1.528 (0.972–2.401); 0.066 | 1.594 (1.019–2.493); 0.041 | 1.511 (0.958–2.382); 0.076 |
| RESIDENCE=CGH | 1.925 (1.274–2.907); 0.002 | 1.937 (1.282–2.927); 0.002 | 1.932 (1.280–2.916); 0.002 | 1.865 (1.234–2.819); 0.003 | 1.921 (1.273–2.897); 0.002 | 1.839 (1.213–2.787); 0.004 |
| RESIDENCE=Independent/family | Reference Category | | | | | |
| Disease = yes | 0.751 (0.493–1.144); 0.182 | 0.797 (0.564–1.125); 0.197 | 0.879 (0.629–1.229); 0.451 | 1.270 (0.914–1.764); 0.155 | 1.200 (0.504–2.857); 0.681 | 1.259 (0.893–1.775); 0.190 |
| Disease = no | Reference Category | | | | | |
| Wave=5 | 0.767 (0.508–1.158); 0.206 | 0.784 (0.519–1.185); 0.248 | 0.787 (0.520–1.191); 0.257 | 0.725 (0.476–1.105); 0.134 | 0.777 (0.515–1.174); 0.231 | 0.743 (0.491–1.126); 0.161 |
| Wave=4 | 3.224 (1.681–6.184); 0.000 | 3.277 (1.708–6.289); 0.000 | 3.273 (1.705–6.283); 0.000 | 3.045 (1.581–5.863); 0.001 | 3.239 (1.689–6.211); 0.000 | 3.101 (1.615–5.954); 0.001 |
| Wave=3 | 0.634 (0.416–0.967); 0.034 | 0.640 (0.419–0.976); 0.038 | 0.640 (0.419–0.977); 0.039 | 0.593 (0.386–0.911); 0.017 | 0.627 (0.411–0.955); 0.030 | 0.604 (0.394–0.924); 0.020 |
| Wave=2 | 2.156 (1.282–3.623); 0.004 | 2.137 (1.272–3.591); 0.004 | 2.152 (1.281–3.615); 0.004 | 2.063 (1.225–3.474); 0.006 | 2.143 (1.276–3.601); 0.004 | 2.103 (1.251–3.535); 0.005 |
| Wave=1 | Reference Category | | | | | |

Supplementary Table 2. GLMM results for utilisation of emergency services across 5 waves controlled for the demographic predictors and chronic conditions

| **Variable** | **Hypertension** | **Lung disease** | **Mental health** | **Stroke** | **Neurological disease** | **Eye disease** |
| --- | --- | --- | --- | --- | --- | --- |
| GENDER = female | 1.062 (0.859 – 1.313); 0.578 | 1.060 (0.859 – 1.309); 0.586 | 1.059 (0.856 – 1.308); 0.599 | 1.059 (0.857 – 1.309); 0.595 | 1.044 (0.849 – 1.282); 0.684 | 1.071 (0.865 – 1.326); 0.527 |
| GENDER = male | Reference Category | | | | | |
| AGE = 65+ | 1.499 (1.102 – 2.039); 0.010 | 1.468 (1.080 – 1.995); 0.014 | 1.503 (1.105 – 2.044); 0.009 | 1.415 (1.038 – 1.930); 0.028 | 1.516 (1.122 – 2.048); 0.007 | 1.537 (1.126 – 2.097); 0.007 |
| AGE = 50–64 | 1.197 (0.938 – 1.527); 0.148 | 1.188 (0.932 – 1.514); 0.165 | 1.194 (0.937 – 1.523); 0.152 | 1.185 (0.929 – 1.512); 0.171 | 1.168 (0.918 – 1.485); 0.206 | 1.207 (0.946 – 1.541); 0.131 |
| AGE < 50 | Reference Category | | | | | |
| Level of intellectual disability = severe/profound | 0.846 (0.622 – 1.150); 0.285 | 0.854 (0.629 – 1.160); 0.312 | 0.846 (0.622 – 1.150); 0.286 | 0.857 (0.629 – 1.165); 0.325 | 0.753 (0.557 – 1.018); 0.065 | 0.851 (0.625 – 1.159); 0.306 |
| Level of intellectual disability = moderate | 0.845 (0.648 – 1.103); 0.216 | 0.849 (0.652 – 1.106); 0.225 | 0.842 (0.646 – 1.099); 0.205 | 0.853 (0.653 – 1.113); 0.241 | 0.804 (0.620 – 1.042); 0.099 | 0.848 (0.649 – 1.108); 0.228 |
| Level of intellectual disability = mild | Reference Category | | | | | |
| Down Syndrome = yes | 1.126 (0.859 – 1.474); 0.390 | 1.116 (0.853 – 1.460); 0.422 | 1.127 (0.857 – 1.481); 0.392 | 1.118 (0.854 – 1.464); 0.419 | 1.173 (0.902 – 1.526); 0.235 | 1.166 (0.884 – 1.536); 0.277 |
| Down Syndrome = no | Reference Category | | | | | |
| RESIDENCE = residential setting | 1.927 (1.372 – 2.708); 0.000 | 1.921 (1.369 – 2.694); 0.000 | 1.935 (1.370 – 2.732); 0.000 | 1.875 (1.334 – 2.636); 0.000 | 1.724 (1.233 – 2.409); 0.001 | 1.950 (1.386 – 2.743); 0.000 |
| RESIDENCE = CGH | 1.474 (1.074 – 2.022); 0.016 | 1.476 (1.078 – 2.022); 0.015 | 1.474 (1.071 – 2.028); 0.017 | 1.461 (1.065 – 2.004); 0.019 | 1.409 (1.033 – 1.921); 0.030 | 1.486 (1.082 – 2.041); 0.014 |
| RESIDENCE = Independent/family | Reference Category | | | | | |
| Disease = yes | 0.998 (0.987 – 1.008); 0.725 | 1.366 (0.963 – 1.937); 0.081 | 0.987 (0.804 – 1.213); 0.903 | 2.010 (1.261 – 3.206); 0.003 | 1.809 (1.480 – 2.212); 0.000 | 0.858 (0.671 – 1.097); 0.222 |
| Disease = no | Reference Category | | | | | |
| Wave | 1.286 (1.103 – 1.498); 0.001 | 1.285 (1.103 – 1.496); 0.001 | 1.283 (1.101 – 1.495); 0.001 | 1.279 (1.098 – 1.490); 0.002 | 1.262 (1.084 – 1.469); 0.003 | 1.292 (1.108 – 1.506); 0.001 |
| Wave³ | 0.993 (0.984 – 1.001); 0.099 | 0.993 (0.984 – 1.001); 0.096 | 0.993 (0.984 – 1.001); 0.102 | 0.993 (0.984 – 1.002); 0.107 | 0.993 (0.984 – 1.002); 0.118 | 0.993 (0.984 – 1.001); 0.095 |

| Variable | Heart disease | Endocrine disease | Joint disease | Gastro-intestinal disease | Cancer | Multimorbidity |
| --- | --- | --- | --- | --- | --- | --- |
| GENDER = female | 1.061 (0.858 – 1.311); 0.587 | 1.066 (0.861 – 1.320); 0.555 | 0.994 (0.806 – 1.225); 0.953 | 1.046 (0.848 – 1.289); 0.676 | 1.064 (0.860 – 1.316); 0.569 | 1.010 (0.820 – 1.245); 0.922 |
| GENDER = male | Reference Category | | | | | |
| AGE = 65+ | 1.471 (1.081 – 2.001); 0.014 | 1.505 (1.105 – 2.049); 0.009 | 1.328 (0.978 – 1.804); 0.069 | 1.450 (1.069 – 1.966); 0.017 | 1.481 (1.088 – 2.016); 0.013 | 1.368 (1.007 – 1.859); 0.045 |
| AGE = 50–64 | 1.186 (0.929 – 1.512); 0.170 | 1.200 (0.940 – 1.531); 0.144 | 1.137 (0.892 – 1.447); 0.299 | 1.183 (0.929 – 1.507); 0.172 | 1.191 (0.933 – 1.519); 0.161 | 1.145 (0.898 – 1.459); 0.274 |
| AGE < 50 | Reference Category | | | | | |
| Level of intellectual disability = severe/profound | 0.846 (0.622 – 1.150); 0.285 | 0.845 (0.621 – 1.150); 0.283 | 0.813 (0.602 – 1.100); 0.179 | 0.760 (0.558 – 1.036); 0.082 | 0.854 (0.627 – 1.162); 0.315 | 0.778 (0.574 – 1.055); 0.106 |
| Level of intellectual disability = moderate | 0.833 (0.639 – 1.087); 0.178 | 0.845 (0.647 – 1.102); 0.214 | 0.821 (0.633 – 1.066); 0.139 | 0.812 (0.623 – 1.056); 0.121 | 0.848 (0.650 – 1.107); 0.226 | 0.807 (0.621 – 1.049); 0.108 |
| Level of intellectual disability = mild | Reference Category | | | | | |
| Down Syndrome = yes | 1.099 (0.838 – 1.442); 0.494 | 1.139 (0.864 – 1.502); 0.355 | 1.118 (0.858 – 1.457); 0.407 | 1.142 (0.875 – 1.491); 0.328 | 1.133 (0.864 – 1.484); 0.366 | 1.131 (0.868 – 1.475); 0.361 |
| Down Syndrome = no | Reference Category | | | | | |
| RESIDENCE = residential setting | 1.920 (1.367 – 2.696); 0.000 | 1.933 (1.375 – 2.717); 0.000 | 1.844 (1.319 – 2.579); 0.000 | 1.811 (1.291 – 2.539); 0.001 | 1.928 (1.371 – 2.710); 0.000 | 1.739 (1.238 – 2.443); 0.001 |
| RESIDENCE = CGH | 1.469 (1.072 – 2.015); 0.017 | 1.475 (1.075 – 2.024); 0.016 | 1.452 (1.064 – 1.982); 0.019 | 1.407 (1.028 – 1.926); 0.033 | 1.480 (1.078 – 2.031); 0.015 | 1.355 (0.989 – 1.857); 0.059 |
| RESIDENCE = Independent/family | Reference Category | | | | | |
| Disease = yes | 1.255 (0.945 – 1.668); 0.117 | 0.957 (0.758 – 1.208); 0.711 | 1.697 (1.376 – 2.094); 0.000 | 1.419 (1.161 – 1.734); 0.001 | 1.354 (0.809 – 2.266); 0.249 | 1.639 (1.291 – 2.080); 0.000 |
| Disease = no | Reference Category | | | | | |
| Wave | 1.279 (1.098 – 1.491); 0.002 | 1.288 (1.105 – 1.501); 0.001 | 1.240 (1.064 – 1.444); 0.006 | 1.224 (1.048 – 1.430); 0.010 | 1.285 (1.103 – 1.498); 0.001 | 1.227 (1.053 – 1.431); 0.009 |
| Wave³ | 0.993 (0.984 – 1.002); 0.120 | 0.993 (0.984 – 1.001); 0.095 | 0.994 (0.985 – 1.002); 0.143 | 0.994 (0.986 – 1.003); 0.193 | 0.993 (0.984 – 1.002); 0.109 | 0.994 (0.986 – 1.003); 0.207 |

Supplementary Table 3: *Odds ratios (OR) and 95% confidence intervals (CI) from* GLMM results for utilisation of outpatient services across 5 waves controlled for the demographic predictors and chronic conditions

| **Variable** | **Hypertension** | **Lung disease** | **Mental health** | **Stroke** | **Neurological disease** | **Eye disease** |
| --- | --- | --- | --- | --- | --- | --- |
| GENDER=female | 1.106 (0.900–1.359); 0.339 | 1.105 (0.900–1.357); 0.339 | 1.102 (0.897–1.353); 0.357 | 1.105 (0.899–1.357); 0.343 | 1.093 (0.892–1.340); 0.392 | 1.090 (0.886–1.340); 0.415 |
| GENDER=male | Reference Category | | | | | |
| AGE_CAT=65+ | 1.323 (0.985–1.777); 0.063 | 1.305 (0.971–1.753); 0.078 | 1.319 (0.982–1.771); 0.066 | 1.307 (0.970–1.760); 0.078 | 1.332 (0.994–1.785); 0.055 | 1.276 (0.947–1.717); 0.109 |
| AGE_CAT=50-64 | 1.264 (1.011–1.579); 0.040 | 1.257 (1.006–1.570); 0.044 | 1.259 (1.008–1.573); 0.043 | 1.260 (1.008–1.575); 0.042 | 1.247 (0.999–1.557); 0.051 | 1.246 (0.997–1.558); 0.054 |
| AGE_CAT < 50 | Reference Category | | | | | |
| Level of intellectual disability =severe/profound | 0.860 (0.638–1.160); 0.324 | 0.866 (0.642–1.167); 0.343 | 0.855 (0.634–1.154); 0.306 | 0.863 (0.639–1.164); 0.333 | 0.799 (0.593–1.077); 0.141 | 0.854 (0.633–1.152); 0.301 |
| Level of intellectual disability =moderate | 0.861 (0.666–1.114); 0.256 | 0.863 (0.668–1.116); 0.262 | 0.858 (0.663–1.109); 0.242 | 0.863 (0.667–1.116); 0.262 | 0.835 (0.647–1.077); 0.166 | 0.858 (0.663–1.111); 0.246 |
| Level of intellectual disability =mild | Reference Category | | | | | |
| Down Syndrome = yes | 1.183 (0.910–1.539); 0.210 | 1.179 (0.907–1.532); 0.218 | 1.216 (0.932–1.586); 0.149 | 1.184 (0.910–1.539); 0.209 | 1.220 (0.941–1.583); 0.134 | 1.126 (0.862–1.472); 0.384 |
| Down Syndrome = no | Reference Category | | | | | |
| RESIDENCE=residential setting | 2.175 (1.586–2.983); 0.000 | 2.172 (1.584–2.977); 0.000 | 2.105 (1.528–2.901); 0.000 | 2.164 (1.577–2.970); 0.000 | 2.020 (1.474–2.767); 0.000 | 2.143 (1.562–2.940); 0.000 |
| RESIDENCE=CGH | 1.927 (1.442–2.575); 0.000 | 1.924 (1.441–2.569); 0.000 | 1.874 (1.399–2.512); 0.000 | 1.923 (1.439–2.570); 0.000 | 1.869 (1.402–2.491); 0.000 | 1.908 (1.428–2.550); 0.000 |
| RESIDENCE=independent/family | Reference Category | | | | | |
| Disease = yes | 0.997 (0.986–1.009); 0.624 | 1.260 (0.883–1.799); 0.203 | 1.119 (0.919–1.362); 0.262 | 1.159 (0.721–1.862); 0.543 | 1.484 (1.216–1.812); 0.000 | 1.261 (0.998–1.593); 0.052 |
| Disease = no | Reference Category | | | | | |
| Wave | 1.106 (0.961–1.272); 0.160 | 1.106 (0.961–1.273); 0.159 | 1.104 (0.959–1.270); 0.168 | 1.105 (0.960–1.272); 0.164 | 1.098 (0.954–1.263); 0.192 | 1.101 (0.957–1.267); 0.179 |
| Wave^3 | 0.990 (0.982–0.999); 0.021 | 0.990 (0.982–0.998); 0.020 | 0.990 (0.982–0.999); 0.022 | 0.990 (0.982–0.999); 0.021 | 0.990 (0.982–0.999); 0.021 | 0.990 (0.982–0.998); 0.020 |

| **Variable** | **Heart disease** | **Endocrine disease** | **Joint disease** | **Gastro-intestinal disease** | **Cancer** | **Multimorbidity** |
| --- | --- | --- | --- | --- | --- | --- |
| GENDER=female | 1.107 (0.901–1.361); 0.332 | 1.047 (0.853–1.285); 0.660 | 1.052 (0.857–1.292); 0.628 | 1.091 (0.889–1.339); 0.405 | 1.110 (0.902–1.366); 0.325 | 1.034 (0.844–1.265); 0.750 |
| GENDER=male | Reference Category | | | | | |
| AGE_CAT=65+ | 1.274 (0.947–1.713); 0.109 | 1.260 (0.940–1.688); 0.123 | 1.201 (0.892–1.616); 0.227 | 1.278 (0.952–1.716); 0.102 | 1.279 (0.949–1.722); 0.106 | 1.155 (0.861–1.550); 0.337 |
| AGE_CAT=50-64 | 1.247 (0.998–1.559); 0.052 | 1.218 (0.975–1.520); 0.082 | 1.224 (0.980–1.528); 0.075 | 1.251 (1.001–1.562); 0.049 | 1.251 (1.000–1.564); 0.050 | 1.193 (0.956–1.490); 0.119 |
| AGE_CAT < 50 | Reference Category | | | | | |
| Level of intellectual disability =severe/profound | 0.864 (0.641–1.166); 0.340 | 0.862 (0.641–1.159); 0.326 | 0.836 (0.621–1.125); 0.236 | 0.771 (0.569–1.044); 0.093 | 0.880 (0.650–1.189); 0.404 | 0.759 (0.565–1.020); 0.067 |
| Level of intellectual disability =moderate | 0.839 (0.648–1.086); 0.183 | 0.860 (0.667–1.109); 0.244 | 0.843 (0.653–1.089); 0.191 | 0.827 (0.639–1.070); 0.148 | 0.869 (0.670–1.126); 0.288 | 0.805 (0.624–1.038); 0.094 |
| Level of intellectual disability =mild | Reference Category | | | | | |
| Down Syndrome = yes | 1.117 (0.857–1.455); 0.415 | 1.049 (0.805–1.368); 0.721 | 1.179 (0.908–1.530); 0.216 | 1.202 (0.925–1.561); 0.169 | 1.204 (0.924–1.569); 0.169 | 1.191 (0.921–1.541); 0.183 |
| Down Syndrome = no | Reference Category | | | | | |
| RESIDENCE=residential setting | 2.160 (1.574–2.964); 0.000 | 2.140 (1.565–2.926); 0.000 | 2.113 (1.543–2.892); 0.000 | 2.051 (1.495–2.813); 0.000 | 2.173 (1.582–2.985); 0.000 | 1.864 (1.361–2.553); 0.000 |
| RESIDENCE=CGH | 1.936 (1.449–2.588); 0.000 | 1.904 (1.429–2.536); 0.000 | 1.901 (1.425–2.535); 0.000 | 1.845 (1.381–2.465); 0.000 | 1.942 (1.451–2.599); 0.000 | 1.709 (1.281–2.279); 0.000 |
| RESIDENCE=independent/family | Reference Category | | | | | |
| Disease = yes | 1.744 (1.312–2.318); 0.000 | 1.649 (1.318–2.063); 0.000 | 1.557 (1.259–1.925); 0.000 | 1.436 (1.184–1.741); 0.000 | 2.253 (1.305–3.889); 0.004 | 2.027 (1.639–2.506); 0.000 |
| Disease = no | Reference Category | | | | | |
| Wave | 1.094 (0.950–1.259); 0.213 | 1.094 (0.950–1.258); 0.211 | 1.077 (0.935–1.240); 0.302 | 1.058 (0.917–1.220); 0.440 | 1.106 (0.961–1.273); 0.162 | 1.039 (0.902–1.197); 0.593 |
| Wave^3 | 0.991 (0.983–0.999); 0.035 | 0.990 (0.982–0.999); 0.022 | 0.991 (0.983–0.999); 0.031 | 0.992 (0.983–1.000); 0.046 | 0.991 (0.983–0.999); 0.028 | 0.993 (0.984–1.001); 0.076 |
